# The Role of Non-Coding RNAs in Cytoplasmic Male Sterility in Flowering Plants

**DOI:** 10.3390/ijms18112429

**Published:** 2017-11-16

**Authors:** Helena Štorchová

**Affiliations:** Institute of Experimental Botany of the Czech Academy of Sciences, Rozvojová 263, 16502 Prague, Czech Republic; storchova@ueb.cas.cz

**Keywords:** cytoplasmic male sterility, non-coding RNA, global transcriptome, gene expression, pollen development

## Abstract

The interactions between mitochondria and nucleus substantially influence plant development, stress response and morphological features. The prominent example of a mitochondrial-nuclear interaction is cytoplasmic male sterility (CMS), when plants produce aborted anthers or inviable pollen. The genes responsible for CMS are located in mitochondrial genome, but their expression is controlled by nuclear genes, called fertility restorers. Recent explosion of high-throughput sequencing methods enabled to study transcriptomic alterations in the level of non-coding RNAs under CMS biogenesis. We summarize current knowledge of the role of nucleus encoded regulatory non-coding RNAs (long non-coding RNA, microRNA as well as small interfering RNA) in CMS. We also focus on the emerging data of non-coding RNAs encoded by mitochondrial genome and their possible involvement in mitochondrial-nuclear interactions and CMS development.

## 1. Introduction

In plants, male sterility refers to the inability to generate viable pollen. It is encoded by nuclear genes leading to the genic male sterility (GMS) or by mitochondrial genes interacting with nuclear genes resulting in the development of cytoplasmic male sterility (CMS). Both kinds of male sterility are broadly utilized in agriculture for the production of hybrid crops providing higher yield than inbred parents [1]. The existence of male-sterile lines eliminates the need for laborious sterilization in a long array of crops including rice (*Oryza sativa*), maize (*Zea mays*), wheat (*Triticum aestivum*), sorghum (*Sorghum bicolor*), sunflower (*Helianthus annuus*) and sugar beet (*Beta vulgaris*). Despite more than two centuries of research and high economic importance, CMS mechanisms remain poorly understood. CMS represents a special case of mitochondrial-nuclear interaction, which is regulated at multiple levels. New discoveries highlight the contributions of non-coding RNAs—a genomic “dark matter” [2]—to the complex regulatory network controlling CMS. In this review, I discuss recent observations and evidence for the action of various classes of plant non-coding RNAs in CMS biogenesis and pollen development.

## 2. Mitochondrial CMS Genes and Their Mode of Action

The mitochondrial and nuclear genes involved in CMS biogenesis or associated with CMS are very diverse [3,4]. Mitochondrial CMS genes are often chimeric, comprised of pieces of essential genes or unknown open reading frames) (ORF) [5,6,7,8]. Chimeric CMS genes can be generated by intramolecular recombination events. For example, maize male-sterile Texas (CMS-T), one of the first CMS lines used in agriculture, possesses *T-urf13* gene [9], derived from at least seven recombination events involving *atp6* and *rrn26* mitochondrial genes. Functional copies of *atp6* and *rrn26* remain in another part of the mitochondrial genome [9]. Another example of a mitochondrial CMS gene is a mutation in *cox2* encoding the cytochrome *c* oxidase subunit 2 truncated protein appearance [10]. CMS genes are very diverse not only across angiosperms, but also within species. For example, numerous CMS systems were described in rice [11,12], maize [9,13], and sugar beet [10,14]. Accordingly, we may expect them to employ similarly diverse modes of action.

The precise details how CMS gene expression impairs mitochondria and the pollen development is not known, but several models have been proposed. Mitochondrial CMS genes may code for cytotoxic proteins like URF13 in maize CMS-T [9], or they may cause energy deficiency during energetically highly energetically demanding male (but not female) reproductive development. Many CMS proteins are hydrophobic and could interfere with oxidative phosphorylation (OXPHOS) complexes within inner mitochondrial membrane [14,15,16], which may decrease the ATP production. Another mechanism can be a premature or delayed programmed cell death (PCD) of the tapetum, the innermost cell layer of the anther wall [17], crucial for the pollen development [18]. PCD of the tapetum, which provides nutrients for the pollen maturation, must be properly timed. CMS genes are often transcribed both in vegetative tissues and anthers, but the respective proteins are produced only at a specific time and tissue. Rice CMS-WA [19] provides a clear example: the WA352 protein accumulates only in tapetal cells and only at the microspore mother cell stage, although *WA352* transcripts are constitutively present in all tissues. Accurate spatiotemporal patterning of CMS-associated mitochondrial genome expression requires fine-tuned regulation at transcriptional, post-transcriptional, translational and post-translational levels. This is achieved by employing plethora of transcription factors and regulatory non-coding RNAs which affect transcript longevity and translation efficiency [2,20].

## 3. Restoration of Fertility by Nuclear Genes

The sterility effects of the mitochondrial CMS genes may be inhibited by the nuclear *Restorer of fertility* (*Rf*) genes, which re-enable the development of functional anthers, fertile pollen, and hermaphroditic flowers [21]. The *Rf* genes suppress CMS genes’ sterilizing effect by degrading or cleaving their mRNAs [22,23], or by post-transcriptional modification including the RNA editing [24]. Alternatively, either the translation of CMS-associated transcript may be blocked [19,25], or the CMS protein degraded [26].

The majority of *Rf* genes belong to the large family of *Pentatricopeptide Repeat* (*PPR*) genes [27]. This gene family, highly expanded in flowering plants, controls multiple aspects of the organellar gene expression, including RNA editing, RNA stabilization and processing, and translation initiation. All PPR proteins contain P-type 35 amino acid domains, each of which recognizes a single nucleotide of RNA [28]. The *Rf* genes constitute a specific *PPR* subfamily called *Restorer of fertility-like* (*RFL*), which shows an accelerated evolutionary rate [29], and numerous domain-level recombination events [30]. Mitochondrial CMS genes and *RFL* genes may co-evolve similarly to a host-pathogen system [31].

In keeping with their tremendous diversity, not all *Rf* genes code for PPR proteins. The *Rf2* gene in maize CMS-T encodes mitochondrial aldehyde dehydrogenase and restores male fertility at a metabolic level [32,33]. Another example is the restoration factor *Rf17* in rice, bearing protein sequence similarity with acyl-carrier proteins [34], which restores male fertility by retrograde mitochondrial-nuclear signaling pathway.

Whereas CMS has been well studied in agricultural plants, its occurrence in the remaining species has been under the less attention. CMS forms the basis for the widespread plant reproduction system-gynodioecy, characterized by the co-occurrence of male-sterile (female) and hermaphrodite individuals in the same populations [35]. However, only a handful CMS systems from natural populations were studied at the molecular level [36,37,38,39]. Domestication is associated with a strong selection for beneficial features which also results in the loss of genetic diversity. We may therefore expect an even more diverse collection of CMS-associated genes in the wild than in agricultural species investigated so far.

## 4. Non-Coding RNAs in Pollen Development and CMS

CMS as a specific case of mitochondrial-nuclear interaction is a complex phenomenon which has to be tightly regulated at multiple levels. Its intricacy became apparent with the recent onset of high throughput methods which enabled complete genome and global transcriptome sequencing [40,41,42,43]. Besides long studied transcription factors [44,45], non-coding RNAs (ncRNAs) became to draw attention. 

Non-coding RNAs contain no large open reading frame (ORF) and are therefore presumed not to encode proteins. They comprise well-studied structural RNAs, such as rRNA, tRNA, snoRNA, snRNA etc., and regulatory RNA. The latter are either longer than 200 nt (long non-coding RNA-lncRNA), or shorter (small RNA–sRNA) [20,46]. Among sRNAs, microRNAs (miRNAs) are the known regulators of gene expression at the post-transcriptional level [47,48]. Another subclass of ncRNAs is represented by small-interfering RNAs (siRNAs), which are involved in the defense against viruses and mobile elements [49,50,51]. Next, *trans*-acting small interfering RNAs (ta-siRNAs) are endogenous regulatory elements participating in complex signaling networks [52]. Of all the classes of regulatory RNAs, only the function of miRNAs in CMS biogenesis has been investigated in detail.

## 5. miRNAs

**Figure 1:**
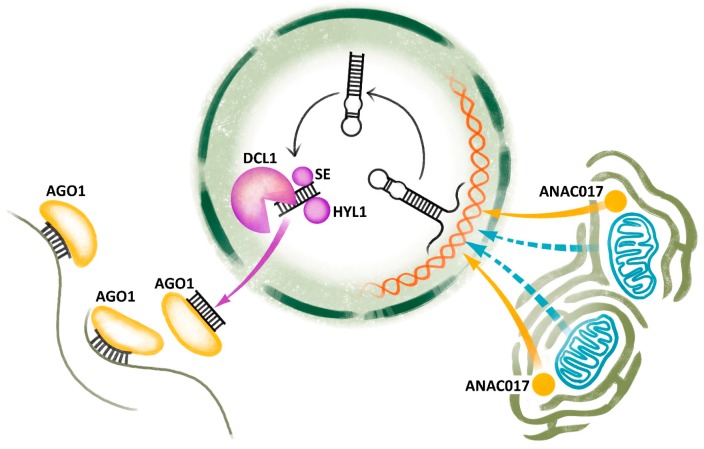
Induction and biogenesis of miRNA during CMS. Mitochondrial biogenesis is altered by the action of cytoplasmic male sterility (CMS)-associated genes sending retrograde signals to the nucleus by means of the NAC transcription factor ANAC017 localized close to endoplasmatic reticulum and/or by other unknown factors. They trigger miRNA gene expression and the production of pri-miRNA, which is subsequently trimmed by the complex containing DICER LIKE1 (DCL1), HYPONASTIC LEAVES1 (HYL), SERRATE (SE) and other proteins in the nucleus. Afterwards, miRNA duplexes are transported to the cytoplasm, where they join ARGONAUTE1 (AGO1), find target mRNAs and initiate its cleavage or translation inhibition by RNA-induced silencing complex (RISC). The figure is based on well-supported model except for blue dashed arrows representing an unknown signal.

miRNAs are small RNAs (about 21 nt) that guide the RNA-induced silencing complex (RISC) to the target transcripts, inducing their cleavage or translational inhibition [47]. They are present in most eukaryotic organisms, but their biogenesis and signaling pathways notably differ between plants and animals [53]. miRNAs are encoded by their own genes at various genomic loci. RNA polymerase II generates a long primary miRNA transcript (pri-miRNA), which contains a hairpin with the miRNA sequence. Plant pri-miRNAs are cut in the nucleus by the complex comprised of RNase DICER LIKE1 (DCL1) and additional proteins e.g., HYPONASTIC LEAVES1 (HYL1) and SERRATE (SE) [54], producing the miRNA duplex. This duplex is transported to the cytoplasm, where it interacts with ARGONAUTE1 (AGO1) to form RISC and to guide it to target genes (Figure 1). Some miRNAs are evolutionary conserved, but many are species-specific [55].

Plant miRNAs control multiple aspects of plant development and stress response, including shoot and root apical development, leaf and trichome development, floral transition and fruit size as well as nutrition-, drought-, salinity- and heat-stress responses [48]. They are also prominent regulators of the pollen development [56]. In the last decade, numerous studies have compared the microtranscriptomes of CMS and fertile lines of agricultural species. (Table 1 and Table S1).

Hundreds of miRNAs have been identified by these studies, many of them belonging to the novel ones. Some miRNAs were differentially expressed between sterile and fertile lines (e.g., 47 in *Brassica juncea* [57]; 42 in cybrid pummelo (*Citrus grandis*) [58]; 87 in *Brassica rapa* CMS-Ogura [59]). Evolutionarily conserved miR156/7a targeting *SQUAMOSA PROMOTER BINDING PROTEIN-LIKE* (*SPL*), which regulates flowering, leaf shape and also tapetum development, was frequently present among differentially expressed miRNAs. Other examples of miRNAs involved in pollen development are miR166 targeting the transcription factor HD-ZIPIII [60], or miR167 targeting *AUXIN RESPONSE FACTOR* (*ARF*) genes, which control anther dehiscence [57]. A broad array of metabolic processes-catabolism of fatty acids [61], sugar transport [61] or inorganic phosphate homeostasis [58] occurring in anthers were affected by the differentially expressed miRNAs. Differentially expressed genes identified by the comparison between the cytoplasmic mRNA-derived transcriptomes of CMS and fertile lines belonged to the similar functional categories as differentially expressed miRNAs. They were involved in starch and sucrose metabolism, amino acid and sulphur metabolism, flavonoid biosynthesis, or pollen development [62,63].

Whereas the impact of mitochondrial CMS genes on the global transcriptome is well described in many crops, the retrograde signal which communicates the mitochondrial impairments to the nucleus of tapetal or pollen cells and triggers diverse cascades of regulatory elements is still elusive. The recently described NAC transcription factor ANAC017 [64] is not the only factor responsible for the mitochondrial retrograde signaling [65] and the mediators communicating between mitochondria and nucleus in CMS are yet to be discovered. In parallel with animal mitochondria [2], we may assume that not only proteins, but also ncRNAs may convey retrograde signals (Figure 1). 

## 6. siRNAs and ta-si RNAs

Unlike miRNAs, siRNAs mediate the silencing of the same genes from which they originate [53]. They are typically 20–24 nt long and are cleaved from a long precursor dsRNA, which may be derived from viruses, transposons or the combination of a sense and antisense transcript. They guide RISC complex to degrade complementary RNA of viral or endogenous origin, or inhibit translation of respective mRNAs. Alternatively, siRNAs mediate de novo modification to form transcriptionally inactive chromatin by recruiting DNA- and histone-modifying enzymes to the specific chromosomal targets [66]. Plant siRNAs may move from cell to cell, but also at longer distance through plasmodesmata or the vascular phloem tissue [53]. They facilitate the communication among individual organs, fine-tuning the response to environmental cues.

The complex interplay between siRNAs and miRNAs is illustrated by the action of ta-si RNAs. They are produced from the transcripts of *TRANS-ACTING SIRNA* (*TAS*) genes which are initially cleaved by the specific miRNAs. Instead being degraded, *TAS* cleavage products are transcribed by the RNA-dependent polymerase and subsequently diced by the DCL4 complex. The resulting siRNAs have a phased pattern, starting at the miRNA cleavage site. They may target the parental or different genes, frequently the members of large gene families [67,68].

The miR173-*TAS1/2*-*PPR* ta-si pathway has been described in *Arabidopsis thaliana* [68,69]. It is triggered by miR173 which initiates the cleavage of *TAS1/2* transcripts and the subsequent production of ta-si RNAs targeting selected *PPR* genes. This pathway is highly conserved across angiosperms [70]. Considering the pervasive influence of *PPR* genes on mitochondrial metabolism, the participation of ta-si RNA in CMS biogenesis is highly plausible, but has not yet been demonstrated. Another example of ta-si pathway which may play a role in the CMS biogenesis is the miR390-*TAS3* module. It may influence pollen development by modulating *ARF* gene expression [68].

## 7. lncRNA

The field of lncRNAs research has expanded and accelerated recently [71,72]. lncRNAs represent the most diverse class of regulatory ncRNA. They are capped and polyadenylated [73,74]; some of them, however, do not contain poly(A) tail [75]. Their modes of action are very diverse. They may be produced as antisense transcripts and to inhibit *sense* transcription (e.g., *COOLAIR*, a cold-induced antisense transcript of the floral inhibitor *FLOWERING LOCUS C* (*FLC*) in *A*. *thaliana* [76]). 

Some lncRNAs influence alternative splicing. They interact with the nuclear speckle RNA-binding protein (NSR), which forms complexes with pre-mRNAs. At least two lncRNAs compete with target pre-mRNAs for NSR and modify their alternative splicing during lateral root formation [77].

The chromatin remodeling caused by lncRNA was also described. The lncRNA *COLDAIR* is transcribed from the intron of the *FLC* gene under cold temperature. It interacts with Polycomb Repressive Complex 2 (PRC2), recruits it to *FLC* and induces *FLC* repression. Silencing the floral inhibitor *FLC* activates flowering in the course of vernalization [78].

lncRNAs often affect the male fertility and the pollen development. Ma and coworkers [79] reported that lnc transcript *zm401* was essential for the tapetum and pollen development in maize. *Long Day Specific Male Fertility Associated RNA* (*LDMAR*) encodes a 1236 nt lncRNA necessary for male fertility under long days. A mutation reducing *LDMAR* transcript levels leads to premature PCD in anthers and male sterility. This example refers to GMS and not to CMS, as no mitochondrial genes are involved in male sterility. 

Some lncRNAs harboring miRNA binding sites function as endogenous target mimics (eTMs) to reduce the repression imposed by miRNAs [80] and to affect the reproductive development–e.g., osa-eTM160 and ath-eTM160 in rice and *A. thaliana*, respectively. They attenuate the repression imposed by miR160 on *ARF*s, which leads to the failure of pollen production [80,81]. 

The crosstalk between lncRNA and siRNA biogenesis has been reported in rice. lncRNAs serve as the source of siRNAs associated with the *MEIOSIS ARRESTED AT LEPTOTENE1 (MEL1)* transcript [82]. MEL1 protein is necessary for the meiotic progress and its loss of function results in aberrant vacuolation of spore mother cells and impaired male fertility. 

Global analyses of long non-coding transcriptomes have greatly expanded our understanding of lncRNA function. The regulatory role of lncRNA in the course of fruit development of hot peppers is particularly known [83]. However, no comprehensive study of lncRNA participation in CMS biogenesis has been published.

## 8. Non-Coding RNAs Encoded by the Mitochondrial Genome 

Whereas knowledge about the regulatory functions of plant nuclear-encoded ncRNAs has been steadily accumulating [47,53,71,72], evidence about the role of ncRNAs encoded by organellar genomes remains sparse. Dietrich and coworkers [84] provided an overview of the functions of organellar ncRNA in plants and animals, including the well-documented regulatory roles of both small and long ncRNAs in animal mitochondria and plant chloroplasts, but they provide only a few candidates of plant mitochondrial ncRNA. Additional examples of mitochondrial ncRNA were described in the comprehensive review on plant organelle biogenesis by Rurek [85]. Small RNAs may be the products of degradation of longer transcripts [86], or may arise due to the relaxed transcription of intergenic regions [75]. As mitochondrial DNA is often transferred to the nucleus [87], a nuclear origin for the already reported mitochondrial ncRNA cannot be excluded.

Ruwe and coworkers [88] described sRNA clusters near the 3′ ends of mitochondrial transcripts in *A. thaliana.* They can stabilize the transcripts or compete for PPR proteins. 

A non-coding mitochondrial transcript about 500 nt long was reported by Holec and coworkers [89] in *A. thaliana*. It carried short stretches of sequence homology with 18S rRNA and tRNA, and exhibited editing sites. No conclusion about its possible function was drawn.

lncRNA accumulated and edited preferentially in male sterile plants (but not in their restored siblings) was documented in bladder campion (*Silene vulgaris*) [90]. Its sequence was not similar to any known sequence in GenBank, it was transcribed from its own promoter. Although it cannot be determined whether this lncRNA is the molecular cause or a consequence of male sterility, it becomes a very first example of mitochondrial ncRNA molecule associated with CMS in plants.

## 9. Future Perspectives

The role of nucleus-encoded miRNAs in CMS has been addressed by numerous studies, as documented in Table 1. However, the function of other classes of regulatory ncRNAs requires additional investigation. In addition to performing new experiments, existing data should be reexamined for the current insights. For example, data sets utilized for the analyses of miRNAs, may be used to study siRNAs in CMS biogenesis. Similarly, CMS-related transcriptomes constructed from polyA-enriched or rRNA-depleted samples may provide information about novel lncRNAs. Given the near-ubiquity of ncRNA involvement in plant development and stress response [48,53,71], investigating their role in CMS should be a priority.

Whereas a plethora of transcription factors and miRNAs induced by CMS and influencing plant metabolism, including mitochondrial functions, have been reported (Table 1), very little is known about retrograde signaling from mitochondria to nucleus (Figure 1). The application of genome editing and the existence loss-of-function mutations in candidate genes in many species with CMS make it possible to reveal the novel candidate genes involved in retrograde signaling during CMS.

Agricultural species went through genetic bottlenecks in the course of domestication which decreased their genetic variation in many genomic regions. As the studies of CMS have been performed primarily in crops, many important aspects or features may have been missed. The investigations of plants from natural populations will likely reveal novel characteristics associated with CMS [36,37,90]. 

## Figures and Tables

**Table 1:** The examples of miRNA and their putative target genes differentially expressed between the CMS lines and their maintainers in various crops.

miRNA	Putative Target Genes	Target Gene Functions	References
Maize CMS C48-2			
Zma-miR397c	*Laccase*	Oxidation of phenolic substrates	
Zma-miR601	*Flavin-containing monooxygenase* (*FMO*)	Auxin biosynthesis	[61]
	*Enoyl-CoA hydratase*	Catabolism of fatty acids	
Zma-miR604	*Monosaccharide transport protein 2 (STP2*)	Uptake of glucose from callose degradation	
*Brassica juncea* hybrid			
miR156a	*SPL* transcription factors	Floral transition, tapetum development	[57]
miR167a	*Auxin response factor* (*ARF6/ARF8*)	Anther dehiscence	
miR319a	*TCP* transcription factors	Floral induction	
miR395a	*ATP sulphurylase (APS)*	Sulphur metabolism	
Rice MeixiangA			
osa-miR528-3p	*F-box containing protein*	Proteolytic turnover through proteasome	[42]
osa-miR1432-5p	*Metal cation transporter*	Cation homeostasis	
osa-miR2118c	*NBS-LRR*	Disease-resistance related proteins	
*Brassica oleracea* Bo01-12A			
bol-miR157a	*SPL* transcription factors	Floral transition, tapetum development	[40]
bol-miR171a	*SCARECROW-like* (*SCL*) transcription factor	GA mediated action	
bol-miR172	*APETALA2* (*AP2*) transcription factor	Floral transition	
bol-miR824	MADS-box transcription factor-like	Plant development	
*Brassica rapa* CMS-Ogura			
bra-miR157a	*SPL* transcription factors	Floral transition, tapetum development	[59]
bra-miR158-3p	*PPR*-*RFL*	RNA metabolism in organelles	
bra-miR159a	*MYB81* transcription factor	Flowering	
bra-miR164a	*CUP SHAPED COTYLEDON 1*	Meristem development	
bra-miR172a	*APETALA2* (*AP2*) transcription factor	Floral transition	
bra-miR5712	*VACUOLAR ATP SYNTHASE SUBUNIT A*	Male gametophyte development	
bra-miR5716	*Zinc finger* transcription factor	Drought stress response	
bra-miR6030	*CC*-*NBS*-*LRR*	Disease-resistance related proteins	
*Glycine max* NJCMS1A			
gma-miR166a-3p	*HD-ZIPIII* transcription factor	Vascular nad cell wall development	[60]
gma-miR169b	*Nuclear factor Y* (*NF-YA*) transcription factor	Flowering	
gma-miR171a	*SCARECROW-like* (*SCL*) transcription factor	GA mediated action	
gma-miR394b-5p	*F-box protein*	Proteolytic turnover through proteasome	
gma-miR395c	*Sulphate transporter 2.1-like*	Sulphur metabolism	
gma-miR396k-5p	*bHLH79* transcription factor	Floral development	
gma-miR397a	*Laccase*	Oxidation of phenolic substrates	
gma-miR408c-3p	*Plastocyanin-like*	Copper metabolism	
*Raphanus sativus* CMS-WA			
miR-158b-3p	*PPR*-*RFL*	RNA metabolism in organelles	[43]
miR161	Mechanosensitive channel of small conductance-like 10 (*MSL10*)	Mechanosensitive ion channel, cell death induction	
miR395a	putative *F-box/kelch-repeat* (*KFB*)	Proteolytic turnover through proteasome	
Pummelo cybrid line			
cga-miR156a.1	*SPL* transcription factors	Floral transition, tapetum development	[58]
cga-miR399a.1	*UBC* (ubiquitin-conjugating E2 enzyme)	Phosphate (P_i_) homeostasis	
cga-miR827	*Basic leucine zipper* (*bZIP*)	Pollen and flower development	

## Supplementary Materials

Supplementary materials can be found at www.mdpi.com/1422-0067/18/11/2429/s1.
